# Screening for Trauma in Pediatric Primary Care

**DOI:** 10.1007/s11920-020-01183-y

**Published:** 2020-09-05

**Authors:** Brooks Keeshin, Kara Byrne, Brian Thorn, Lindsay Shepard

**Affiliations:** Department of Pediatrics, Center for Safe & Healthy Families, University of Utah, 81 N. Mario Capecchi Dr., Salt Lake City, UT 84113 USA

**Keywords:** Trauma, Traumatic stress, Adverse childhood experiences (ACES), Trauma screening, Pediatric primary care

## Abstract

**Purpose of Review:**

Provided the high prevalence of trauma exposure in childhood as well as the risk for morbidity, this article examines evidence, a recommended approach, and key implementation factors relevant to screening for trauma in pediatric primary care.

**Recent Findings:**

A standardized approach to trauma screening is possible, but previous attempts have relied heavily upon exposure screening and failed to guide an individualized response specific to the impact of trauma on the child and family. Trauma screening tools for pediatric primary care should be brief and inform the care response based on screening for trauma exposure, traumatic stress symptoms, functional impact, and suicidality.

**Summary:**

Clinicians should use trauma screening to (1) identify if the child has any ongoing risk of harm and report where required; (2) determine risk of suicidality and respond appropriately; (3) assess need for evidence-based trauma treatment based on symptoms and functional impact; and (4) provide a skill or guidance targeting the most severe or pressing traumatic stress symptoms.

## Introduction

As many as 80% of children are exposed to a potentially traumatic experience in childhood. A potentially traumatic experience, or trauma, is “a significant event or experience that causes or threatens harm to one’s emotional and/or physical well-being” [1••]. Examples include sexual or physical abuse, family or community violence, life-threatening accidents or medical diagnoses, natural disasters, war, and terrorism. There is a large and compelling body of evidence that demonstrates that exposure to potentially traumatic experiences in childhood is associated with both short-term and long-term morbidity [2]. Furthermore, the type, chronicity, and number of exposures all likely contribute to the risk of ongoing emotional and behavioral challenges after traumatic events.

Given the broad range of potential exposures that can occur over a multitude of developmental periods in childhood, the impact of trauma can be quite varied—and has the potential to impact all domains of functioning, cognition, and emotional regulation. However, there are a number of symptoms that are predictably seen in many youth who continue to be impacted by at least one potentially traumatic experience. These symptoms are likely related to changes in the function and coordination between the sympathetic and autonomic nervous systems and together make up the constellation of symptoms that can form the diagnosis of posttraumatic stress disorder (PTSD). More broadly, these symptoms are commonly called traumatic stress, “intense fear and stress in response to a potentially traumatic experience, including disturbed sleep, difficulty paying attention and concentrating, anger and irritability, withdrawal, repeated and intrusive thoughts, and/or extreme distress when confronted by reminders of the trauma” [3].

Provided the high prevalence of trauma exposure in childhood as well as the risk for short- and long-term morbidity, this article will examine evidence, a recommended approach, and key implementation factors relevant to screening for child trauma exposure and traumatic stress in pediatric primary care.

## Evidence for Screening for Adversities, Trauma Exposure, and Traumatic Stress Symptoms

Interest in detecting adverse childhood experiences (ACEs) developed rapidly once clear links were established between ACEs and many of the most common and costly adult health problems [4]. The possibility of improving the lives of children and reducing societal healthcare costs by identifying and responding to ACEs has motivated calls for increased screening (www.acesaware.org). However, others have cautioned about the potential for unintended outcomes and significant expenditures with little benefit [5••, 6••]. Early identification of certain adversities may be an avenue to effective treatment that can mitigate the negative impact, but effective treatments are not always accessible or available (at any cost), and the potential to magnify perceived stigma or highlight racial or social inequities without meaningful responses is high in many situations. For example, ACEs include some experiences, such as parental incarceration, poverty, exposure to community violence, and parental depression or substance abuse, that may be disproportionately represented in some racial, ethnic minority and/or immigrant communities. Highlighting the increased presence of adverse experiences among youth in these communities without acknowledging historical trauma and systemic inequities or providing evidence-based or meaningful intervention creates a risk that parents and families will feel blamed for the harmful effects of circumstances they may have little or no ability to control. Further, merely knowing that an adverse experience has occurred informs clinicians little about how the experience(s) has negatively impacted a particular child. Intrapersonal factors (such as intelligence) and interpersonal factors (such as caregiver/family support, community/institutional support) promote resilience and facilitate positive coping. Simply highlighting existing ACEs may, in fact, lead clinicians to miss important opportunities to bolster support for patients who are functioning at a relatively high level in spite of adverse experiences by validating their efforts. The value of healthcare screening is highest when the condition is common yet not routinely detected, when knowing about the condition may lead to a different treatment response, and when effective treatments are available.

While screening for adverse experiences or trauma exposure alone is not strongly supported in the literature, screening for traumatic stress symptoms among those with a history of potentially traumatic experiences can identify children who may benefit from evidence-based trauma treatment. Research shows that different children having essentially the same upsetting experience may be impacted in different ways [7]. Some will have few or no symptoms of traumatic stress; some will have symptoms that diminish over several weeks; and some may have long-lasting emotional, behavioral, and cognitive difficulties that affect multiple domains of life as a result of the experience(s). Some will experience suicidality [8]. Particularly for those with severe symptoms or acute problems, such as suicidality, the impact can be pervasive and damaging while limiting normative developmental experiences that support healthy functioning. Screening for symptoms and functional difficulties provides meaningful data to guide interventions that range from crisis services and evidence-based treatments (EBT) for significantly impacted youth to anticipatory guidance and validation of the youth and caregivers’ efforts in children who are doing well. Efficient, targeted screening for severity and pattern of symptoms can enhance the primary care treatment relationship by facilitating an individualized response that is more likely to be perceived as helpful by a particular patient/family.

Several validated measures for trauma-specific symptoms have been developed. Some measures, such as the Trauma Symptom Checklist for Children/Trauma Symptom Checklist for Young Children (TSCC/TSCYC), the UCLA PTSD Reaction Index (UCLA PTSD-RI), and the Child PTSD Symptom Scale (CPSS), may be too long and provide more detail than necessary in the primary care setting. Recently, the UCLA Brief Screen for Trauma and PTSD (UCLA Brief Screen), an 11-item child traumatic stress screening measure, was derived from the full UCLA PTSD-RI and validated against other well-supported measures of child PTSD [9]. While it does not provide the comprehensive traumatic stress data of the larger instrument, the UCLA Brief Screen accurately identifies youth who are most likely to have ongoing traumatic stress-related problems and thus are most appropriate for further trauma-informed assessment and evidence-based trauma therapy [10]. Its brief format is also well-suited for pediatric primary care screening.

### Traumatic Stress Dictates Appropriate Intervention

Over the past generation, significant progress has been made in the effort to develop effective empirically supported mental health treatments for child traumatic stress. One key example is Trauma-Focused Cognitive Behavioral Therapy (TFCBT) for children and adolescents, shown to have superior outcomes to other methods in more than 20 randomized controlled clinical trials, conducted in different settings (including Europe and Africa) and with diverse populations and varied life circumstances [11, 12]. It is a structured, short-term psychotherapeutic treatment model that effectively improves trauma-related outcomes for children/teens and their caregivers. TFCBT addresses affective, cognitive, and behavioral problems; promotes optimal support at home; strengthens parenting skills; and reduces child and caregiver distress about the child’s traumatic experiences. With support from the National Child Traumatic Stress Network (NCTSN) and others, there has been widespread dissemination of TFCBT over the past 15 years. Over 120,000 mental healthcare professionals have participated in a 2-day TFCBT training event, and certified therapists can be found in all 50 states (www.tfcbt.org). While TFCBT has been recognized by the Substance Abuse and Mental Health Services Administration (SAMHSA) as a Model Program (www.tfcbt.org), other specialized child trauma treatment models are showing emerging evidence of improved outcomes compared with typical child therapy approaches. Mental health therapists with specialized trauma treatment training are more available and accessible now than ever in the past. Among the children who have had potentially traumatizing experiences, those with elevated symptoms of traumatic stress are significantly more likely to have positive treatment outcomes if they are connected with mental health therapists who use specialized trauma-focused evidence-based treatment, such as TFCBT. Ideally, trauma and traumatic stress screening would be used to identify children and teens who are most likely to benefit from trauma-focused evidence-based treatment and to prompt referral from pediatric primary care.

## A Recommended Approach to Trauma Screening

Similar to screening for other common pediatric conditions, it is essential that a trauma screening process be feasible and provides meaningful clinical data that is directly applicable to the care of the child and family. Similarities between trauma screening and other behavioral health screens (i.e., PHQ) include the systematic detection of symptoms with a validated tool to differentiate sick versus well and highlight degrees of severity. However, in contrast to other behavioral health screens, trauma screening also requires the detection and appropriate response to lived experiences (trauma exposure), some of which may continue to pose an ongoing risk to the child if not addressed. Table 1 highlights some of the common groupings of trauma exposure and trauma symptoms important for primary care trauma screening and response. Ultimately, the most important objectives of trauma screening in pediatric primary care are to (1) identify and respond to child trauma exposure (including safety) and (2) identify and respond to child reactions and symptoms. Initially, most guidance for trauma screening in pediatric primary care has been dominated by screening solely for trauma exposure, limited by the sole availability of lengthy, behavioral health measures for traumatic stress, and frustrated by the omission of guidance directing the care response. More recent recommendations by the American Academy of Pediatrics highlight the need for identifying and responding to traumatic stress symptoms as part of a comprehensive, trauma-informed approach in primary care [1••, 13••].

**Table 1:** Identifying and responding to trauma exposure and trauma reactions/symptoms

Trauma Exposure	
Identify	Respond
Child maltreatment and family violence	Report abuse or exposure to violence when indicated to keep child safe
Special populations (e.g., youth in foster care, refugee youth)	Coordinate and collaborate between varying systems of care
Familial challenges (e.g., a hurt or sick caregiver, community violence/crime)	Identify support for impacted family members
Secondary adversities (e.g., loss of housing, food insecurity, educational displacement)	Connect to case management to support housing, financial, legal, or other needs
Trauma reactions/symptoms	
Identify	Respond
Suicidality	Assess for risk using a validated process such as the Columbia Suicide Severity Rating Scale
Functional impairment	Provide letters and connect to case management when needed (e.g., letter to school for accommodations, consideration of 504/IEP)
Minimal traumatic stress symptoms	Validate resilience, provide anticipatory guidance, and systematically screen for symptoms
Moderate or severe traumatic stress symptoms	Provide education, skills, or techniques targeted at specific symptoms; refer to evidence-based, trauma-focused therapists for assessment and treatment

In recognition of the childhood and lifetime burden of trauma for children as well as its under-identification and underdeveloped response in healthcare, our center was funded by SAMHSA to develop a standardized process for the identification and management of pediatric traumatic stress in primary care (https://utahpips.org) [14]. As part of a care process model (CPM) for *The Diagnosis and Management of Traumatic Stress in Pediatric Patients*, the goal of the Pediatric Traumatic Stress Screening Tool is to identify children at risk for traumatic stress and inform the primary care clinical response (available for download at either: https://intermountainhealthcare.org/ckr-ext/Dcmnt?ncid=529796906 or https://utahpips.org). The process provides meaningful information about specific area(s) of difficulty to guide the clinician in determining the most helpful next step for the child/family. Decision support guides a clinical response that is targeted to safety and symptoms, directs anticipatory guidance and follow-up for those who are doing well, and identifies those who may benefit from evidence-based trauma assessment and treatment.

The Pediatric Traumatic Stress Screening Tool is a 15-question tool with decision support and can be used as part of a general screening protocol, either alone or in combination with other screeners (such as in combination with the PHQ-A or as part of case finding where safety or behavioral health concerns already exist). The primary components of the tool include a stem that defines a potentially traumatic exposure and then asks two open-ended questions about recent or past exposures. Next, 12 trauma-specific questions are asked, providing detailed frequency information on sleep issues, intrusive and arousal challenges, and difficulties with avoidance and negative cognitions and mood. The validated UCLA Brief Screen comprises 11 of the 12 items. In the model, an additional trauma symptom question was added to provide a two-question sleep subscale. Finally, in youth who are not already being screened for suicidality, the PHQ-A question #9 is included as the first step of a suicide screen, with all youth who screen positive receiving the short version of the Columbia Suicide Severity Rating Scale, either as a questionnaire or verbally, to further categorize suicide risk.

**Fig. 1:**
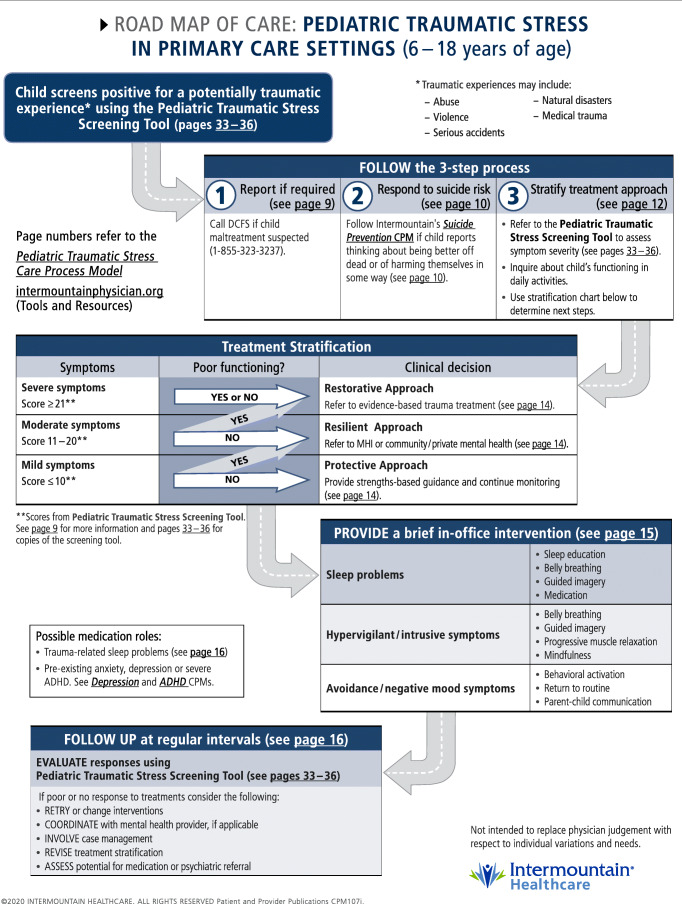
Decision support for pediatric traumatic stress in primary care settings

The decision support highlights 4 primary responses for youth who screen positive for a potentially traumatic experience (Fig. 1):

1. Identify if the child has any ongoing risk of harm from the reported traumatic events and report where required
2. Determine risk of suicidality and respond appropriately
3. Assess ongoing impact of traumatic experiences based on symptom burden and functional impairment, providing referrals for evidence-based trauma-focused assessment and treatment to those youth who would most benefit
4. Provide a skill or guidance to directly target the most severe or pressing constellation of traumatic stress symptoms.

Built within the model are tools and the data necessary to make informed referral decisions that are specific to the needs of the patient, as well as lay the ground work for collaborative care with a therapist who can provide evidence-based trauma assessment and treatment. Since the UCLA Brief Screen is derived from the full UCLA PTSD-RI for DSM 5, when mental health therapists use the full measure as part of their trauma-informed assessment, there is congruency between the measures. Finally, in a pediatric setting, the UCLA Brief Screen administration can be repeated, so that the pediatrician can monitor progress as needed.

### Screening Versus Case Finding

Trauma screening and response must be adapted to the needs of the specific pediatric population. For screening, pediatric clinics could choose to universally screen at all well child visits over the age of 5 or based on other standardized criteria. However, for clinics who choose not to universally screen, the same tool can be used for all designated “mental health” visits. In this context, the pediatrician is no longer screening, but rather using the tool as part of a trauma-informed approach to the evaluation of other common pediatric behavioral health concerns such as mood, worries, and behavior problems. In this context, the trauma screen assures that the emotions or behaviors of concern (i.e., a chief complaint of possible ADHD or depression) would not be better explained by a trauma reaction, thus warranting a different approach to treatment.

## Implementation

Implementation efforts are critical to the success of traumatic stress screening and response within primary care. Specifically, as informed by the CFIR implementation model, preparing and working within the inner setting (within clinic factors) and outer setting (external policies and community partners) are essential [15, 16] (Table 2).

**Table 2:** Implementation model for the inner and outer settings

Inner setting implementation efforts	
Identify a clinic champion for trauma-informed care	
Educate team on child trauma, traumatic stress, and evidence-based trauma treatment	
Prepare and train staff in screening and responding to trauma	
Refine clinic knowledge on trauma and resources as screening begins	
Embed a data feedback loop for iterative improvements to trauma detection and response	
Outer setting implementation efforts	
Educate health system administration on importance of trauma-informed care	
Identify and categorize specific referral resources for evidence-based trauma treatment	
Engage with mental health clinics and schools for comprehensive trauma-informed systems	
Advocate for additional trauma-focused resources if/when community capacity is not sufficient to meet the identified population of youth who warrant evidence-based trauma assessment and treatment	

### Inner Setting

The inner setting is the culture, workflow, and staff of the clinic, including both clinical and nonclinical staff. Specific implementation efforts include identifying a clinic champion(s), advocating for trauma screening and response, preparing and training clinicians and staff, providing ongoing technical assistance, and, where possible, installing data feedback loops and mechanisms to identify and make needed adaptations.

Identifying a clinic champion(s) is generally the first step. The clinic champion is a care team member who will advocate for trauma screening and response, learn and study the model, coordinate training(s), plan and pilot the workflow, and encourage adoption across clinicians and staff.

To advocate for trauma screening and response, the clinic champion may offer or arrange presentations on child trauma, traumatic stress, and trauma screening and response. Essentially, it is important for the entire team to understand what trauma and traumatic stress are and how trauma screening and response will help them respond effectively to the needs of patients and families. Didactic components might also review the trauma reactions that can mimic commonly considered disorders in children, and evidence-based treatments can help improve traumatic stress and other trauma-associated symptoms [13••].

After general training on trauma and child traumatic stress, provided there is some interest or buy-in, the clinic champion can provide or arrange an overview or training in a selected trauma screening approach. For example, if the clinic champion or care team selects *The Diagnosis and Management of Traumatic Stress in Pediatric Patients: A Care Process Model* (https://utahpips.org) [14], the following items would be available for review:

- The Pediatric Traumatic Stress Care Process Model
- The Pediatric Traumatic Stress Screening Tool
- The 3-step process (Fig. 1)
- Brief in-office interventions
- Approaches to identify resources for evidence-based trauma treatment and therapists accessible to clinic patients*
- Discussion with clinicians and staff, proposing potential workflow adaptations, sharing relevant resources in the community, and identifying potential barriers to implementation

Following training and if he/she is not already doing so, the clinic champion can pilot the proposed workflow and provide ongoing technical assistance to support uptake of the new screening process.

When possible, integrating a data feedback loop into the process can encourage clinic-wide motivation to increase or continue to screen for child trauma. General components to consider include incorporating data collection measures into existing workflow, establishing a regular timeline for reporting, and identifying data points to inform clinic processes (e.g., duration of clinic visits, number of positive screeners, common clinician decisions). Meeting with clinicians and staff a few months into implementation will help identify facilitators and barriers to screening, and if perceived barriers such as added time are raised by clinicians or staff, having objective data on duration of visits and benefits to families based on increased detection and referral will help address those concerns.

### External Setting

Obtaining support from outside key players is a critical step in supporting implementation. You could consider these key players in two groups, clinic administrative leaders and relevant community partners. Given the potential for training and changes to workflow, proactively engaging with administrative leaders prior to implementation is an opportunity to make the clinical justification for adding trauma screening while presenting anticipated barriers to implementation and proposed options to mitigate those challenges. This allows for administrative leaders not only to provide support to the initiative but also to provide feedback on implementation strategies, increasing the likelihood of additional resources/support as needed.

As clinicians screen for and respond to child trauma, they will need to make referrals to evidence-based trauma services near the families they serve. In order to do so, working with community partners to identify and build on community capacity in referral sources for evidence-based trauma therapy is critical prior to full-scale implementation. Not all communities will have adequate or equitable access to evidence-based trauma therapy. Early engagement with community providers not only identifies therapists already providing services but also facilitates communication to support alignment between clinic referral practices driven by the ability to deliver evidence-based trauma treatments. This opens the door for collaborative efforts to build community capacity to support evidence-based providers (e.g., increase community therapist’s access to training and support in delivering evidence-based trauma therapies). Functionally, ongoing maintenance of a resource list includes the name, contact information, wait times, and insurances accepted for therapists practicing evidence-based trauma-informed modalities.

### Evaluation

Evaluation, especially on top of implementation, can feel like a lot to take on. That being said, evaluation can describe the work being done, highlight improvements, increase motivation and buy-in, and inform focused adaptations. Simple tally methods might track clinic or clinician progress in implementing trauma screening, such as number of eligible children screened or number of children provided resources or referrals for traumatic stress. Results could be tallied weekly or monthly and posted to a clinic bulletin board.

Some clinics may opt for a more rigorous evaluation of trauma screening, including applying for Maintenance of Certification (MOC) credit for quality improvement. It could be helpful to collaborate with outside evaluators and data warehouse experts to help you develop meaningful and efficient evaluation design, data collection, and analysis strategies. Some key questions to consider include:

- How many clinicians are screening for trauma?
- Of the patients in your clinic who are eligible to be screened for trauma, how many were actually screened?
- What safety issues are being identified through trauma screening (i.e., child abuse, suicidality)?
- How many or what proportion of children screened for trauma are receiving a care response?
- Are children with moderate and high symptoms for traumatic stress provided referrals to therapy or, specifically, evidence-based trauma treatment?
- On any or all of the above items, how has performance changed over time (e.g., after 6 or 12 months of implementation)?

## Conclusions

Due to the high prevalence of trauma exposure in childhood as well as the risk for short- and long-term morbidity, there have been increasing calls and efforts to screen for trauma in pediatric primary care. A standardized approach to trauma screening is possible, but previous attempts have relied heavily upon exposure screening and failed to guide an individualized response specific to the impact of the trauma on the child and family. There is strong evidence as to the effectiveness of evidence-based trauma treatments in resolving or significantly decreasing child traumatic stress [11, 12, 13••]. For trauma screening in pediatric primary care, trauma screening tools must be brief and inform the care response based on screening for trauma exposure, traumatic stress symptoms and severity, and suicidality. Clinicians can use trauma screening to:

1. Identify if the child has any ongoing risk of harm from the reported traumatic events and report where required
2. Determine risk of suicidality and respond appropriately
3. Assess need for evidence-based trauma-focused assessment and treatment based on symptoms and functional impact
4. Provide a skill or guidance to directly target the most severe or pressing constellation of traumatic stress symptoms

A clinic champion and other planned implementation efforts are critical to the success of traumatic stress screening and response within primary care. Evaluation or simple quality improvement tally methods can track and motivate screening progress. Ultimately, trauma screening in pediatric primary care can not only identify children exposed to potentially traumatic experiences in childhood but also respond to their needs, providing an opportunity to prevent and/or disrupt negative, long-term sequelae of trauma.

## Data Availability

The Care Process Model (CPM) for *The Diagnosis and Management of Traumatic Stress in Pediatric Patients* is available open-access at https://intermountainhealthcare.org/ckr-ext/Dcmnt?ncid=529796906 or https://utahpips.org/. For additional information on the CPM or other resources for pediatric traumatic stress, please contact the corresponding author.
